# Redefining the gap in Aboriginal health: from deficit to cultural connection

**DOI:** 10.1016/j.lanwpc.2024.101176

**Published:** 2024-11-10

**Authors:** Ted Fields, Warren Foster, Brett J. Biles, Aryati Yashadhana

**Affiliations:** a School of Population Health, University of New South Wales, Sydney, NSW, Australia; b Centre for Primary Health Care & Equity, University of New South Wales, Sydney, NSW, Australia; c Deputy Vice-Chancellor Indigenous Division, University of New South Wales, Sydney, NSW, Australia; d School of Social Sciences, University of New South Wales, Sydney, NSW, Australia

## Abstract

This article is written from the perspectives of a Yuwaalaraay/Gamilaraay cultural knowledge holder and a Yuin Djirringanj cultural knowledge holder from New South Wales, Australia. It explores the concept of cultural health, and the need to shift towards centring culture in every aspect of Aboriginal health and wellbeing. The three elements of cultural health are discussed as Country which includes lands, waters, skies, and all entities within; people, and their freedom, and ability to express and maintain continuing connections to culture and Country; and culture which encompasses identity, language, and knowledges, and is maintained and strengthened through active connection to Country, and cultural practices. Cultural health is out of balance due to invasion and ongoing colonisation which translates to the differences in health and social outcomes we see represented in ‘the gap’, fails to acknowledge or centre our cultural health, and remains a challenge in making significant progress in health and social outcomes. It is reflected in many of the unmet or receding targets. To improve Aboriginal health and wellbeing, data exploring the different aspects of ‘the gap’ in cultural health from a strength-based approach, as opposed to the gap in deficit and disease is required.

This commentary is based on a discussion between Ted Fields, a Yuwaalaraay and Gamilaraay cultural leader and knowledge holder originally from Walgett, New South Wales (NSW), and Warren Foster, a Yuin-Djirringanj cultural leader, knowledge holder and songman from Wallaga Lake NSW. This article is written from their perspectives. While we do use the term ‘Aboriginal’ in this article to explain the terminology of ‘the gap’, we would like to acknowledge it as a colonised and homogenising term; and advocate shifting to the use of cultural group names as identifiers.

For our people to be healthy, we must have access to our Country and our culture. Culture cannot exist as we know it without Country, and people who are from that Country and practice that culture form the connection in-between. These three elements are reflected in our creation stories, they are interdependent and fundamentally place-based, and when functioning as they are supposed to, as they did for 60,000 plus years, our cultural health is strong. If any one of those elements are out of balance, the others can become weak or sick, and for balance and health to return, each element plays a role. The way these elements intertwine can be exemplified through the cultural and ceremonial practice of burning Country. By bringing Country back to a culturally healthy landscape, the health and wellbeing of the people also benefit,^1^ through a cultural ceremony of reciprocal care and relationship. These elements were central in creation and are central now.

In the context of invasion and ongoing colonisation, our people's cultural health is out of balance, which translates to the differences in health and social outcomes we see represented in ‘the gap’. The gap measures the difference in health and social outcomes between Aboriginal and non-Aboriginal people in Australia, shaping policies, funding, and research.^2^ While it is important to understand these differences, the gap is deficit, disease, and problem based, meaning everything that stems from it (including funding and resources), is also framed in the same way (e.g. body part funding). The gap as we know it fails to acknowledge or centre our cultural health, which remains a challenge to making significant progress in health and social outcomes; and is reflected in many of its unmet or receding ‘targets’.^2^

Therefore, we also know what is preventing us from returning our cultural health to a state of balance. This is what we refer to here as ‘the gap’ in cultural health. This idea differs from the gap in health and social outcomes because it centres cultural ways of being, knowing, and doing that are strengths-based.

The first element of cultural health, Country, incorporates lands, waters, skies, and all entities that exist within these.^3^ Country is also known to us as our (Earth) Mother, *Gunimaa* (Yuwaalaraay/Gamilaraay) or *Mirriwan Minga* (Yuin-Djirringanj), whereby a reciprocal relationship of care with the people, which happens through cultural practices, defines both its health and the people's health. Presently, our people have little to no control or authority over, or access to Country due to colonised laws and systems that prioritise private and state ownership. Systems that were put in place to empower us have failed. For example, the Aboriginal Land Rights Act (NSW) has produced little material outcomes, with almost 70% of claims remaining undetermined since it was introduced in 1983.^4^ Proving a continued cultural connection to acquire land under the Native Title Act (Commonwealth), is even more challenging.^5^ Therefore, the most crucial ‘gap’ in maintaining and improving our cultural health, is authority, control, and unmitigated access to our Country—without this progress will remain stagnant.

The second element is our people's freedom, and ability to express and maintain continuing connections to Country. Colonisation, racism, genocide, and displacement from ancestral Country have weakened cultural connections for some of us. Many people are wanting to reconnect, and we are seeing the health and healing benefits that come from reconnection.^6^, ^7^, ^8^ However, funding and resourcing, as well as individual financial and social pressures create barriers to maintaining and restoring these connections; forming another key aspect of the cultural health gap. In this special collection, we discuss cultural camps as one promising format to facilitating such connection, however as knowledge holders who facilitate these camps, we see resourcing as an ongoing challenge to their delivery.^7^

The third cultural health element is our culture. Culture encompasses identity, language, and knowledges, and is maintained and strengthened through our people's active connection to Country, and expressed through cultural practice. If our culture is healthy, by being renewed, practiced, and passed on to the younger generation, then the health of Country and the people also benefit. Yet many barriers lie in the way of our people accessing cultural knowledges and language, particularly the opportunity to learn these in-place on-Country, as we always did. This forms another fundamental aspect of the cultural health gap, whereby the recognition, resourcing and support given to cultural leaders, knowledge holders and language practitioners in their tireless (and often unpaid) work in cultural regeneration is key.

The only data relative to cultural health that is being collected on a regular and national scale is the use of our languages. While this is important, it is only one aspect of the elements discussed here and reinforces the romanticisation of our culture to those who dictate policy and resourcing. To shift to strengths-based approaches to improving the health and wellbeing of our people, data exploring the different aspects of ‘the gap’ in cultural health discussed here, as opposed to the gap in deficit and disease, are urgently needed.

## Contributors

TF—Conceptualisation; Writing—original draft; Writing—review & editing; Supervision.

WF—Conceptualisation; Writing—original draft; Writing—review & editing; Supervision.

BJB—Conceptualisation; Writing—review & editing; Funding acquisition.

AY–Conceptualisation; Writing—original draft; Writing—review & editing; Funding acquisition.

## Declaration of interests

None declared.
